# Supplementary material for: Sputum culture reversion in longer treatments with bedaquiline, delamanid, and repurposed drugs for drug-resistant tuberculosis
Source: Nat Commun. 2024 May 9;15:3927. doi: 10.1038/s41467-024-48077-8 (PMC11082252; doi:10.1038/s41467-024-48077-8)
Supplement: Supplementary file 2 — Reporting Summary [file 41467_2024_48077_MOESM2_ESM.pdf]

nature portfolio

Corresponding author(s): Molly Franke

Last updated by author(s): Mar 22, 2024

Reporting Summary

Nature Portfolio wishes to improve the reproducibility of the work that we publish. This form provides structure for consistency and transparency in reporting. For further information on Nature Portfolio policies, see our Editorial Policies and the Editorial Policy Checklist.

Statistics

For all statistical analyses, confirm that the following items are present in the figure legend, table legend, main text, or Methods section.

n/a	Confirmed	
☐	☒	The exact sample size (*n*) for each experimental group/condition, given as a discrete number and unit of measurement
☐	☒	A statement on whether measurements were taken from distinct samples or whether the same sample was measured repeatedly
☐	☒	The statistical test(s) used AND whether they are one- or two-sided<br>*Only common tests should be described solely by name; describe more complex techniques in the Methods section.*
☐	☒	A description of all covariates tested
☐	☒	A description of any assumptions or corrections, such as tests of normality and adjustment for multiple comparisons
☐	☒	A full description of the statistical parameters including central tendency (e.g. means) or other basic estimates (e.g. regression coefficient) AND variation (e.g. standard deviation) or associated estimates of uncertainty (e.g. confidence intervals)
☐	☒	For null hypothesis testing, the test statistic (e.g. *F*, *t*, *r*) with confidence intervals, effect sizes, degrees of freedom and *P* value noted<br>*Give P values as exact values whenever suitable.*
☒	☐	For Bayesian analysis, information on the choice of priors and Markov chain Monte Carlo settings
☒	☐	For hierarchical and complex designs, identification of the appropriate level for tests and full reporting of outcomes
☐	☒	Estimates of effect sizes (e.g. Cohen's *d*, Pearson's *r*), indicating how they were calculated

Our web collection on statistics for biologists contains articles on many of the points above.

Software and code

Policy information about availability of computer code

Data collection	Data were collected prospectively as part of routine care using standardized forms. The endTB EMR (Database MySQL) was developed based on an open-source system called ‘Bahmni’ (https://www.bahmni.org) in collaboration with an independent software company. The endTB EMR was designed to capture detailed longitudinal patient-level data across countries and freely available for use by other MDR-TB programs or projects (install and set up: https://github.com/endtb/endtb-config; https://github.com/endtb/endtb-config/wiki/Deployment-Steps).
Data analysis	Open source code (survival package v 3.2-7, Therneau, 2020) for R was used to perform univariable and multivariable Cox regression analyses in R version 4.1.2.

For manuscripts utilizing custom algorithms or software that are central to the research but not yet described in published literature, software must be made available to editors and reviewers. We strongly encourage code deposition in a community repository (e.g. GitHub). See the Nature Portfolio guidelines for submitting code & software for further information.

Data

Policy information about availability of data

All manuscripts must include a data availability statement. This statement should provide the following information, where applicable:

- Accession codes, unique identifiers, or web links for publicly available datasets
- A description of any restrictions on data availability
- For clinical datasets or third party data, please ensure that the statement adheres to our policy

Some of the data included in this analysis are managed in countries governed by the European Union General Data Protection Regulation (GDPR). The data contain sensitive and potentially identifying information and cannot be sufficiently anonymized to meet GDPR standards and retain their utility. Pseudoanonymized data will be made available within 2 weeks upon request to an MSF Medical Director at endTB.ClinicalTrial@paris.msf.org, and execution of a data sharing agreement or alternate means that allows assurance that principles of GDPR regulations will be met.

Research involving human participants, their data, or biological material

Policy information about studies with human participants or human data. See also policy information about sex, gender (identity/presentation), and sexual orientation and race, ethnicity and racism.

Reporting on sex and gender	Sex was included in the study design as baseline demographic information while gender was not collected (gender was not routinely collected across all countries during the study period). Sex was determined by clinicians recorded in the EMR. Disaggregated sex data are available in the source data. Out of 1,286 participants in our sample 440 (34%) were female. We did not perform sex-based analysis due to a small number of reversion events which would have made it difficult to interpret study findings.
Reporting on race, ethnicity, or other socially relevant groupings	We did not include data on race, ethnicity, or other socially relevant groupings as these concepts have different meanings across the countries included in the study.
Population characteristics	Population characteristics in the study included basic demographic characteristics such as age and sex. Median age for participants was 35 (25th-75th percentile: 27.0-47.0) and 34.2% of the participants were female. Comorbitidities such as diabetes, cardiovascular disease, cancer, renal dysfunction, psychiatric illness, HIV, hepatitis B and C were collected.
Recruitment	Patients consecutively initiating tuberculosis treatment in participating clinics across 17 countries were invited to enroll for this study. During the informed consent process, study staff communicated potential risks, and were informed they may withdraw consent at any time without any impact on their treatment and follow-up. Selection bias may have occurred if those who refused study participation had different risks of reversion than those who accepted participation.
Ethics oversight	This study was approved by the Partners Healthcare Human Research Committee (Boston, MA, USA), the MSF Ethics Review Board (Geneva, Switzerland), IRD Institutional Review Board (Karachi, Pakistan) and in all 17 enrolling countries by local ethics committees or IRBs.

Note that full information on the approval of the study protocol must also be provided in the manuscript.

Field-specific reporting

Please select the one below that is the best fit for your research. If you are not sure, read the appropriate sections before making your selection.

☒ Life sciences ☐ Behavioural & social sciences ☐ Ecological, evolutionary & environmental sciences

For a reference copy of the document with all sections, see nature.com/documents/nr-reporting-summary-flat.pdf

Life sciences study design

All studies must disclose on these points even when the disclosure is negative.

Sample size	No sample -size calculation was performed. All available data were analyzed.
Data exclusions	Patients treated in Democratic People's Republic of Korea (n=45), where comorbidity screening and follow-up procedures differed, were excluded from the analysis. Hepatitis B infection was excluded from the multivariable model because only one individual with baseline hepatitis B infection experienced reversion.
Replication	Not relevant to study as it is an observational study.
Randomization	Not relevant to study as it is an observational study.
Blinding	Not relevant to study as it is an observational study.

Reporting for specific materials, systems and methods

We require information from authors about some types of materials, experimental systems and methods used in many studies. Here, indicate whether each material, system or method listed is relevant to your study. If you are not sure if a list item applies to your research, read the appropriate section before selecting a response.

Materials & experimental systems

n/a	Involved in the study	
☒	☐	Antibodies
☒	☐	Eukaryotic cell lines
☒	☐	Palaeontology and archaeology
☒	☐	Animals and other organisms
☐	☒	Clinical data
☒	☐	Dual use research of concern
☒	☐	Plants

Methods

n/a	Involved in the study	
☒	☐	ChIP-seq
☒	☐	Flow cytometry
☒	☐	MRI-based neuroimaging

Clinical data

Policy information about clinical studies

All manuscripts should comply with the ICMJE guidelines for publication of clinical research and a completed CONSORT checklist must be included with all submissions.

Clinical trial registration	NCT03259269 (registration of the observational study).
Study protocol	Khan, U., Huerga, H., Khan, A.J. et al. The endTB observational study protocol: treatment of MDR-TB with bedaquiline or delamanid containing regimens. BMC Infect Dis 19, 733 (2019). https://doi.org/10.1186/s12879-019-4378-4
Data collection	Patients were enrolled in 17 countries: Armenia, Bangladesh, Belarus, Democratic People's Republic of Korea (DPRK), Ethiopia, Georgia, Haiti, Indonesia, Kazakhstan, Kenya, Kyrgyzstan, Lesotho, Myanmar, Pakistan, Peru, South Africa and Vietnam. Patients included in this analysis initiated treatment between 1 April 2015 and 30 September 2018 at an endTB site. Study participants' clinical data was collected under the supervision of the National Tuberculosis Program or local implementing parters at each clinical site, without additional interventions or procedures.
Outcomes	Primary outcomes for efficacy (favorable and unfavorable end of treatment outcomes) and safety (incidence and outcome of clinically relevant adverse events). Secondary outcomes for efficacy (frequency and time to relapse, frequency of conversion, frequency of and time to reversion, and frequency of recurrence-free survival).

Plants

Seed stocks	*Report on the source of all seed stocks or other plant material used. If applicable, state the seed stock centre and catalogue number. If plant specimens were collected from the field, describe the collection location, date and sampling procedures.*
Novel plant genotypes	*Describe the methods by which all novel plant genotypes were produced. This includes those generated by transgenic approaches, gene editing, chemical/radiation-based mutagenesis and hybridization. For transgenic lines, describe the transformation method, the number of independent lines analyzed and the generation upon which experiments were performed. For gene-edited lines, describe the editor used, the endogenous sequence targeted for editing, the targeting guide RNA sequence (if applicable) and how the editor was applied.*
Authentication	*Describe any authentication procedures for each seed stock used or novel genotype generated. Describe any experiments used to assess the effect of a mutation and, where applicable, how potential secondary effects (e.g. second site T-DNA insertions, mosiacism, off-target gene editing) were examined.*
